# An antigenic epitope of influenza virus nucleoprotein (NP) associated with polymeric forms of NP

**DOI:** 10.1186/1743-422X-5-37

**Published:** 2008-02-29

**Authors:** Elena N Prokudina, Nataly Semenova, Valery Chumakov, Lothar Stitz

**Affiliations:** 1 The D.I. Ivanovsky Institute of Virology, Gamaleya str. 16, Moscow, Russia; 2 Friedrich-Loeffler-Institut, D-72076 Tubingen, Germany

## Abstract

Intracellular influenza virus nucleoprotein (NP) is characterized by a high efficiency of homo-polymers formation, however their antigenic structure is still incompletely known. Herein, we report that RNase-resistant intracellular NP homo-polymers have a highly ordered conformational antigenic epitope, which depends on inter-subunit interactions of monomeric NPs. Our studies have shown that in radioimmunoprecipitation (RIPA) intracellular NP polymers bind mAb N5D3 and RNase does not prevent their mAb binding. In contrast to NP polymers, NP monomeric subunits, obtained by thermo-dissociation of NP polymers, fail to bind the mAb N5D3 in RIPA. At the same time, the *in vitro *concentration of thermo-denatured monomeric NPs in both soluble and immobilized forms results in NP-NP association, accompanied by renaturation of the N5D3 epitope. The same results were detected by Western blotting, where the pre-denatured NP monomers were concentrated on nitrocellulose into a single 56 kDa band, which then caused NP-NP self-association as well as N5D3 epitope renaturation. Thus, the *in vitro *renaturation of N5D3 epitope is markedly dependent on NP monomers concentration.

The results obtained suggest that *in vivo *formation and *in vitro *renaturation of the N5D3 epitope depend on inter-subunit interactions of monomeric NPs and NP-NP interactions influence the antigenic structure of the influenza virus NP polymers.

## Findings

It is known that intracellular nucleoprotein (NP) is capable of self-associating to form large RNA-free homo-polymeric complexes [1,2], which are morphologically similar to the intact viral RNP [3-5]. We have previously shown that numerous types of RNase resistant thermo-sensitive NP polymers are detected in influenza virus infected MDCK cells [6-8]. After heating, NP polymers are dissociated exclusively into NP monomeric subunits. It is also known that protein-protein interactions induce conformational changes at interfaces of subunits. As a result, those polymerizing proteins may acquire new biological properties, including the exposure of new conformational epitopes [9,10]. The antigenic structure of intracellular influenza virus NP homo-polymers is still unknown.

In the present study, we have analyzed the total intracellular influenza virus NP polymers and demonstrated *in vivo *formation and *in vitro *renaturation of the antigenic epitope depending on NP-NP association.

Influenza A/Duck/Ukraine/63(H3N8) and MDCK (Madin Darbin Canine Kidney) cells were used. The NP was detected using rabbit anti-NP polyclonal antibody [1] and anti-NP mAbs.

For mAb generation, the intracellular influenza virus NP isolated from chorionallantoic membranes of embryonated chicken eggs infected with A/FPV/Rostock/34(H7N1) influenza virus was used. Intracellular NP was purified by immunoaffinity chromatography and isoelectric focusing [1,11]. For the present study, a monoclonal antibody against NP designated mAb N5D3 was selected.

For metabolic labeling of the infected cells, [^35^S] methionine (50 μCi/ml) was introduced into the medium for 1 hr at 5 hrs p.i. Before SDS-PAGE analysis the cell lysate was divided into two portions: one portion was left unheated to preserve NP polymers, and the other was heated for 40 min at 70°C (or 3 min at 100°C) to dissociate NP polymers into NP monomeric subunits. Both unheated and pre-heated portions were analysed by RIPA, Dot-blot assay and Western blotting.

RIPA, Western blot and Dot-blot assays were carried out as described [6,12].

In the first series of experiments, we compared the mAb N5D3 binding capacity of intracellular NP polymers with their solubilized monomeric subunits using RIPA and Western blot.

**Figure 1:**
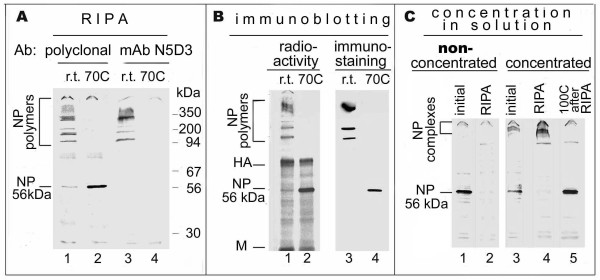
**The capacity of polymeric and monomeric NP to bind mAb N5D3**. A) RIPA. Radiolabeled cytosol of infected cells was divided into unheated (r.t) portion, containing NP polymers and the heated (70° C) portion, containing NP 56 kDa monomers as a result of NP polymers dissociation. Both unheated and pre-heated portions were subjected to RIPA using polyclonal anti-NP Abs or mAb N5D3. SDS-PAGE of the immunoprecipitates obtained by RIPA using polyclonal Abs (lanes 1, 2) and mAb N5D3 (lanes 3,4). B) Immunolotting. Radiolabeled unheated (1,3) and pre-heated (2,4) cytosols were subjected to SDS-PAGE, followed by Western blot, including electro-transfer onto nitrocellulose membrane, autoradiography and immunodetection using mAb N5D3. Autoradiography (lanes 1, 2) and immunostaining using mAb N5D3 (lanes 3,4) of membrane containing blotted proteins. C) Renaturation of N5D3 epitope caused by self-association of the concentrated soluble NP monomers. The non-concentrated m-NP before RIPA (lane 1) and after immunosorption by RIPA using mAb N5D3 (lane 2). The concentrated soluble self-associated m-NP (as described in the text) before RIPA (lane 3) and after immunosorption by RIPA using mAb N5D3 (lane 4). The aliquot of RIPA immunoprecipitate shown in lane 4 was heated at 100°C for 3 min before SDS-PAGE (lane 5). The samples shown in lanes 1–4 were not additionally pre-heated before SDS-PAGE.

As shown in Fig. 1A the polyclonal antibodies (Abs) reacted in a RIPA with both polymeric NPs, which were present in the unheated cytosol (lane 1), and monomeric 56 kDA NPs, which were a result of thermo-dissociation of NP polymers (lane 2). As also shown NP polymers were recognized by mAb N5D3 in unheated cytosol (lane 3). The pre-treatment of cytosol with RNase did not influence the ability of NP polymers to bind mAb N5D3 (not shown). In contrast to NP polymers, the soluble 56 kDa NP monomers formed after thermo-dissociation of NP polymers were not recognized by mAb N5D3 in a RIPA (lane 4). A trivial explanation could be that the conformational N5D3 epitope is present not only in polymeric NPs but also in monomeric NP subunits, but as a result of the heating process, this epitope is denatured and destroyed. If this assumption is correct, the 56 kDa NP monomers transferred onto nitrocellulose after heating and denaturing SDS-PAGE should not be recognized by mAb N5D3 in a Western blot, as they were not recognized in the heated cytosol by a RIPA (shown in Fig. 1A, lane 4).

To study the mAb N5D3 binding ability of monomeric NPs in a Western blot, the unheated and pre-heated radiolabeled cytosols were subjected to denaturing SDS-PAGE followed by transfer onto nitrocellulose membrane. Fig. 1B shows the pattern of the total intracellular proteins detected on nitrocellulose by autoradiography (lanes 1, 2), and the same proteins immunostained using the mAb N5D3 (lanes 3, 4). The immunostaining results showed that in the unheated sample mAb N5D3 recognized the immobilized NP-polymers (lane 3). RNase treatment of immobilized NP polymers did not decrease their mAb N5D3 binding capacity (not shown). It is also shown in Fig. 1 that in contrast to RIPA (Fig. 1A, lane 4), the thermo-denatured 56 kDa NP monomers were efficiently recognized by mAb N5D3 in Western blot analysis (Fig. 1B, lane 4).

One of the reasons for the differences in immunodetection of monomeric NP between RIPA and Western blot may be the difference in concentrations of monomeric NPs in the two analyses. According to a calibration curve of Coomassie staining (not shown), ~1 μg of monomeric NPs in a single 56 kDa band (shown in Fig. 1B, lanes 2 and 4) localized on a membrane in a volume of about 1 mm^3 ^in the Western blot (5 mm × 2 mm × 0.1 mm corresponding to length × width × depth of the 56 kDa NP band). However, before electrophoresis, the same 1 μg of monomeric NPs was present in 50 mm^3 ^of initial cytosol. Therefore, ~0.02 μg/mm^3 ^of monomeric NP was in the initial cytosol detected in a RIPA and ~1 μg/mm^3 ^of monomeric NPs was in a 56 kDa band detected in a Western blot. The ~50-fold increase of NP concentration on the membrane in a Western blot leads to shortening in the intermolecular distances, and this presumably promotes NP-NP association, accompanied by N5D3 epitope renaturation.

In further experiments, the dependence of N5D3-epitope renaturation on the concentration of monomeric NPs was studied in both soluble and immobilized forms of NPs. For this aim the solution containing the radiolabelled polymeric NPs (about 1000 ng/ml) was obtained by N5D3-mAb-mediated affinity chromatography [11] using the unheated cytosol. The purified polymeric NPs were divided into an unheated portion containing only polymeric NPs (p-NP) and a heated portion containing only monomeric NPs (m-NP).

To concentrate the monomeric NPs in a soluble form, the pre-heated solution was placed in a dialysis bag and the volume was reduced 10-fold by covering the bag with dry Sephadex G-200. The concentrated solution was then stored at +4°C for 72 hrs with shaking to provide the additional NP-NP interactions. The reduced volume was then reconstituted to the initial volume and RIPA analysis was carried out. As shown in Fig. 1C (lane 1), only 56 kDa monomeric NPs were detected in the pre-heated non-concentrated solution of m-NP. These non-concentrated monomeric NPs were not recognized by N5D3 mAb in a RIPA (Fig. 1C, lane 2). However, after the procedures of m-NP concentration, some complexes appeared in a stacking gel (Fig. 1C, lane 3), which were recognized by N5D3 mAb in a RIPA (lane 4) and dissociated after heating into NP monomers (lane 5). The data obtained indicated that as a result of m-NP concentration in solution, the intermolecular distance is reduced, which causes the formation of NP-NP complexes, promoting renaturation of the N5D3 epitope (Fig. 1C, lane 4).

**Figure 2:**
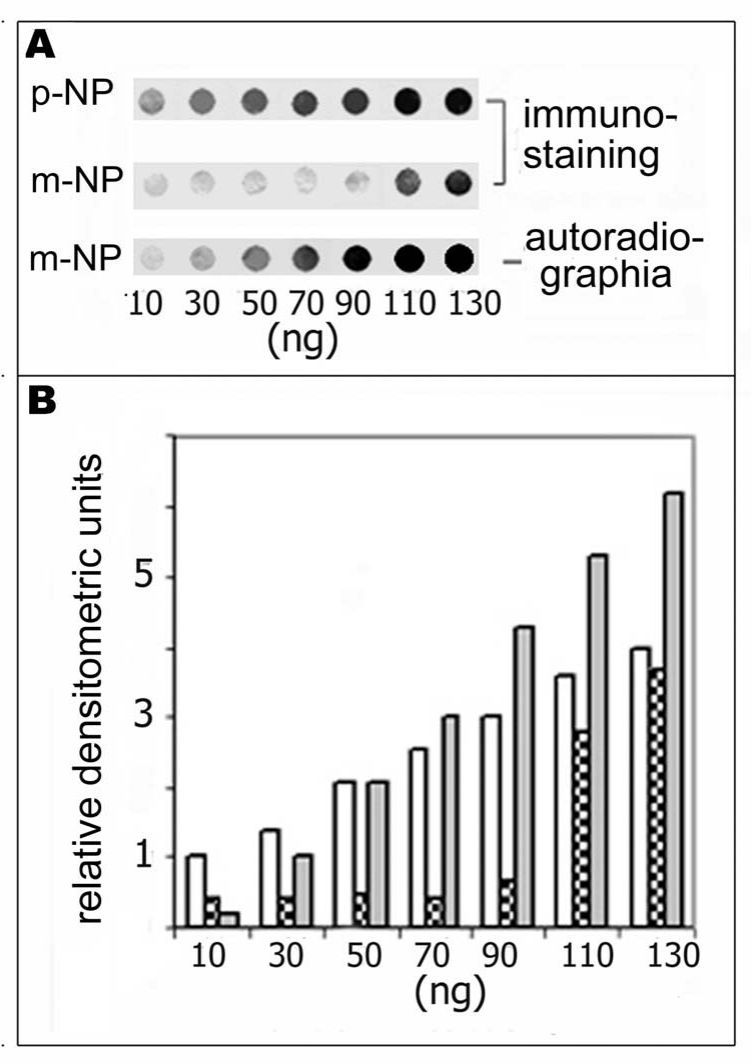
**Concentration dependence of mAb-N5D3-binding capacity of monomeric NPs immobilized on membrane**. The solutions containing the radiolabeled polymeric (p-NP) and monomeric (m-NP) NPs were repeatedly spotted in a volume of 10 μl onto nitrocellulose. As a result, the indicated increasing amount of NP were loaded into the dots. The dots were then subjected to immunostaining (A, upper and middle row), autoradiography (A, lower row) and densitometric analysis (B). Densitometric analysis of the spots (B) shown in Fig. 2A: white columns – immunodetection of p-NP corresponding to the upper row ; dotted columns – immunodetection of m-NP corresponding to the middle row; grey columns – autoradiographs of m-NP corresponding to the lower row.

To concentrate the immobilized NP, solutions containing either polymeric (p-NP) or monomeric (m-NP) NPs were loaded onto a nitrocellulose membrane (~10 ng NP in 10 μl) in increasing amounts, using repeated spotting onto the same sites. The resulting spots were arranged in horizontal rows and contained NP concentrations ranging from 10 ng to 130 ng (Fig. 2A).

All dots spotted onto the membrane were subjected to immunostaining using the N5D3 mAb, autoradiography and densitometry. It was shown that NP polymers (p-NP) exhibited an approximately linear concentration dependence of their N5D3 mAb binding efficiency (Fig. 2A, upper row; Fig. 2B, white columns). In contrast, the monomeric NPs (m-NP) demonstrated a strong non-linear concentration dependence of their mAb binding capacity (Fig. 2A, middle row; Fig. 2B dotted columns). The radioactivity demonstrated a linear concentration dependence for both monomeric NPs (Fig. 2, lower row and grey columns) and polymeric NPs (not shown). These data suggest that N5D3 epitope renaturation by immobilized monomeric NPs corresponds to a "cooperative" biological phenomenon and is due to NP-NP association.

Taken together the results obtained indicate that the highly ordered conformational antigenic epitope depending on NP-NP association is described in the present study. This suggestion is based on the following observations. Firstly, in a RIPA soluble NP polymers bind mAb N5D3 with high efficiency, whereas soluble NP monomeric subunits, obtained by thermo-dissociation of NP polymers, fail to bind the mAb N5D3. Secondly, the concentration of both soluble and immobilized pre-denatured NP monomers causes NP self-association and restoration of the N5D3-epitope.

The mechanism whereby formation of the N5D3 epitope is dependent on NP-NP association remains a matter of speculation. Most likely, the interaction of NP subunits modifies the conformation of their interfaces and, as a result, the neo-epitope may be exposed as has been described for other polymeric proteins [9,10].

It is known that conformational epitopes are immunodominant in comparison with linear epitopes [13]. Therefore, on the basis of the high efficiency of *in vitro *NP-NP association, one may predict that as a result of immunization with concentrated NPs NP-polymer-specific mAbs may be efficiently generated. Immunization with the influenza virus NP results in a protective effect due to activation of cytotoxic T lymphocytes [14]. Besides, the antibody-dependent protective effect (other than virus neutralization) is also known for influenza virus NP [15] and antigenic epitope depending on NP-NP association probably takes part in this mechanism together with the other epitopes. The results obtained in this report show that NP-NP interactions influence the antigenic structure of the influenza virus NPs. Therefore the oligomeric state of NPs should probably be taken into account when designing influenza vaccines. Thus, in this report we described the phenomenon concerning with the existence of unique antigenic epitope, which depends on NP-NP association and localized in intracellular RNase resistant NP polymers.

## Authors' contributions

EP and NS composed the initial conception, contributed to parts of the experimental work and to data interpretation. VC assisted the experiments as well as data analysis. LS coordinated the research efforts, provided with polyclonal and monoclonal antibodies and revised the manuscript. All authors have read and approved the manuscript.
